# QOI6/416: The Health On the Net Code of Conduct for Medical and Health-related Web sites: Three years on

**DOI:** 10.2196/jmir.1.suppl1.e99

**Published:** 1999-09-19

**Authors:** C Boyer, V Baujard, T Nater, JR Scherrer, RD Appel

**Affiliations:** ^1^Health On the Net Foundation, GenevaSwitzerland

**Keywords:** Quality Assurance, Quality Health Care, Peer Review, Ethics

## Abstract

**Introduction:**

The explosive growth of the World Wide Web has made it more and more urgent to monitor and improve the quality of the information circulating through the Internet. But assessing medical and health information on the Web is a difficult challenge. In recent years, some organisations have been working on this issue. The Health On the Net Foundation (HON), for its part, administers an eight-point Code of Conduct called the HONcode, initiated in 1996. This solution is different from the others. The HONcode does not intend to rate or assess the quality of information provided by a Web site. This article gives a general presentation of the HONcode and its status in 1999, three years after it was launched.

**Methods:**

It defines a set of voluntary rules designed to help a Web developer site practice responsible and to make sure a reader always knows the source and the purpose of the information he or she is reading. These guidelines encourage the authority, complimentarity, confidentiality, proper attribution, justifiability and validity of the medical advice and information provided. Furthermore, sites that subscribe to the HONcode commit themselves to providing transparent information on site sponsorship and clearly separating advertising and editorial content.

**Results:**

In 1998, the number of external links to the HONcode principles page grew by about 250% (e.g., from 2 to 5) . The rate of increase from January through April, 1999, suggests 300% this year. The growth in the number of links to the HONcode matches that of links to of the entire Web site, and is slightly higher than growth in links to HON's MedHunt search engine. Statistical analysis shows that approximately 50% of Web sites linked to the HONcode principles have a commercial domain name, 16% are from European countries, 15% are from non-profit organisations, and 7% are educational sites.

**Discussion:**

This evolution shows a real need of guidelines for the developers of medical and health Web sites and their users. This conclusion is reinforced by the result of an international survey which HON conducted on the Internet in March and April 1999. The HONcode's scope of application is currently limited to publishing policy and ethical aspects. The HONcode does not address issues of quality and the relevance of the health and medical content of a Web page -- and this is a crucial issue for the further healthy development of the Internet in the medical domain. One way to assess and provide reliable medical and health content could be to review medical and health-related Web content by peers. Building on the system adopted by the scientific community decades ago for the paper medium, this Electronic peer review method could cover content as well as ethical and publishing aspects while retaining use of the HONcode display icon on registered sites as an additional reference.

